# Recommended operating room practice during the COVID‐19 pandemic: systematic review

**DOI:** 10.1002/bjs5.50304

**Published:** 2020-06-04

**Authors:** Welsh Surgical Research Initiative (WSRI) Collaborative, T. Abdelrahman, J. Ansell, C. Brown, R. Egan, T. Evans, E. Ryan Harper, R. L. Harries, L. Hopkins, O. James, S. Lewis, W. G. Lewis, O. Luton, K. Mellor, A. G. Powell, D. Robinson, R. Thomas, A. Williams, A. J. Beamish

**Affiliations:** ^1^ School of Surgery Health Education and Improvement Wales Nantgarw UK

## Abstract

**Background:**

The COVID‐19 pandemic poses a critical global public health crisis. Operating room (OR) best practice in this crisis is poorly defined. This systematic review was performed to identify contemporary evidence relating to OR practice in the context of COVID‐19.

**Methods:**

MEDLINE was searched systematically using PubMed (search date 19 March 2020) for relevant studies in accordance with PRISMA guidelines. Documented practices and guidance were assessed to determine Oxford Centre for Evidence‐Based Medicine (OCEBM) levels of evidence, and recommendations for practice within five domains were extracted: physical OR, personnel, patient, procedure, and other factors.

**Results:**

Thirty‐five articles were identified, of which 11 met eligibility criteria. Nine articles constituted expert opinion and two were retrospective studies. All articles originated from the Far East (China, 9; Singapore, 2); eight of the articles concerned general surgery. Common themes were identified within each domain, but all recommendations were based on low levels of evidence (median OCEBM level 5 (range 4–5)). The highest number of overlapping recommendations related to physical OR (8 articles) and procedural factors (13). Although few recommendations related to personnel factors, consensus was high in this domain, with all studies mandating the use of personal protective equipment.

**Conclusion:**

There was little evidence to inform this systematic review, but there was consensus regarding many aspects of OR practice. Within the context of a rapidly evolving pandemic, timely amalgamation of global practice and experiences is needed to inform best practice.

## Graphical abstract

This rapid systematic review was performed to identify contemporary evidence relating to operating room (OR) practice during the COVID‐19 pandemic. Evidence underpinning the review was sparse; however, consensus regarding many aspects of OR practice was apparent.

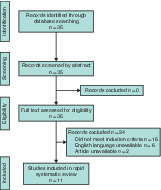

High quality evidence needed

## Abstract (es)

**Antecedentes:**

La pandemia por COVID‐19 plantea una crisis crítica de salud pública a nivel mundial. La mejor práctica en el quirófano en esta crisis está mal definida. Esta revisión sistemática se realizó para identificar la evidencia contemporánea relacionada con la práctica en el quirófano en el contexto del COVID‐19.

**Métodos:**

Se realizó una búsqueda sistemática en Medline usando PubMed (fecha de búsqueda, 19 de marzo de 2020) para seleccionar estudios relevantes de acuerdo con las directrices PRISMA. Las prácticas documentadas y las guías se evaluaron para determinar los niveles de evidencia según la normativa Oxford (Oxford *levels of evidence*, OLE), y se extrajeron las recomendaciones para la práctica en el entorno de cinco ámbitos: físico en el quirófano, personal, paciente, procedimiento y otros factores.

**Resultados:**

Se identificaron 35 artículos, de los cuales 11 cumplían con los criterios de elegibilidad. Nueve artículos constituían opinión de expertos y dos fueron estudios retrospectivos. Todos los artículos provenían del Lejano Oriente (nueve de China, dos de Singapur); ocho de los artículos estaban relacionados con cirugía general. Se identificaron temas comunes en cada ámbito, pero todas las recomendaciones se basaron en bajos niveles de evidencia, mediana de 5 OLE (rango 4 ‐ 5). El mayor número de recomendaciones superpuestas se relacionaban con el ámbito físico del quirófano (*n* = 8) y con factores del procedimiento (*n* = 13). Aunque hay pocas recomendaciones relacionadas con factores del personal, el consenso fue alto en este ámbito, y todos los estudios exigieron el uso de equipos de protección personal.

**Conclusión:**

Hubo poca evidencia para que esta revisión sistemática proporcionara información, pero existió consenso con respecto a muchos aspectos de la práctica en el quirófano. En el contexto de una pandemia en rápida evolución, es preciso aglutinar a tiempo real las estrategias y experiencias globales a fin de poder informar acerca de las mejores prácticas.

## Introduction

Coronavirus has killed thousands since emerging in China in December 2019, and compelled many governments to lock down populations^1^.

Preliminary data from China and Italy regarding the spectrum of severity and fatality vary. China reported that 80 per cent of those infected reported no or mild, 15 per cent severe, and 5 per cent critical symptoms, with a mortality rate ranging from 0·25 to 3·0 per cent^2^, indicating demand for intensive medical intervention for one in five patients. Case fatality is much greater in the vulnerable: patients aged 80 years or more (mortality rate above 14 per cent) and those with coexisting conditions, such as cardiovascular disease (10 per cent) and diabetes (7 per cent)^3^.

From an acute surgical perspective, although some patients may present with an acute abdomen secondary to the viral infection, it is more likely that general surgeons will encounter patients with common acute abdominal pathology and undiagnosed COVID‐19 infection, or those who develop nosocomial COVID‐19 infection while an inpatient with a surgical diagnosis. Guidance on perioperative care of surgical patients with suspected or proven coronavirus infection is slender, and operating room (OR) best practice remains unknown.

The aim of this study was to perform a systematic review of the literature to identify and collate global experience, practice and recommendations relating to OR practice in the context of the COVID‐19 pandemic.

## Methods

A rapid systematic review of published work was conducted using standard rapid review methodology, as outlined by Schünemann and Moja^4^ and in accordance with the PRISMA guidelines^5^.

MEDLINE was searched via PubMed on 19 March 2020 (no date restriction), for articles describing specific practices, or providing recommendations or guidance relating to OR practice, in the context of the COVID‐19 pandemic. No limitation was placed on language or publication type, but non‐English‐language articles without extractable data were excluded. Relevant articles were identified using terms in any field relating to coronavirus (for example, coronavirus, COVID‐19, SARS‐CoV‐2) and the OR (such as operating room, theatre, surgery), and operating room practice (such as preparation, procedures, guidance, advice, practice, recommendations). The full search algorithm is shown in *Appendix* *S1* (supporting information). Further articles were identified by hand search of references and using the PubMed related articles function. Levels of evidence were determined as described by the Oxford Centre for Evidence‐Based Medicine (OCEBM)^6^.

### Data extraction

Nine authors extracted data independently, and two verified a random subsample of seven articles. The following details were extracted: first author, date of publication, study design, country, region, and any description of specific practices, recommendations or guidance in relation to OR practice in the context of the COVID‐19 pandemic. Five domains for data capture were identified *a priori*: the physical OR factors, personnel factors, patient factors, procedure factors, and other considerations.

### Inclusion and exclusion criteria

Articles reporting specific practices, experience, recommendations or guidance in relation to emergency OR practice, in the context of the COVID‐19 pandemic, were included.

Articles were excluded if they did not meet the inclusion criteria, if the full‐text article was unavailable, or if no English version was extractable.

## Results

**Figure 1:**
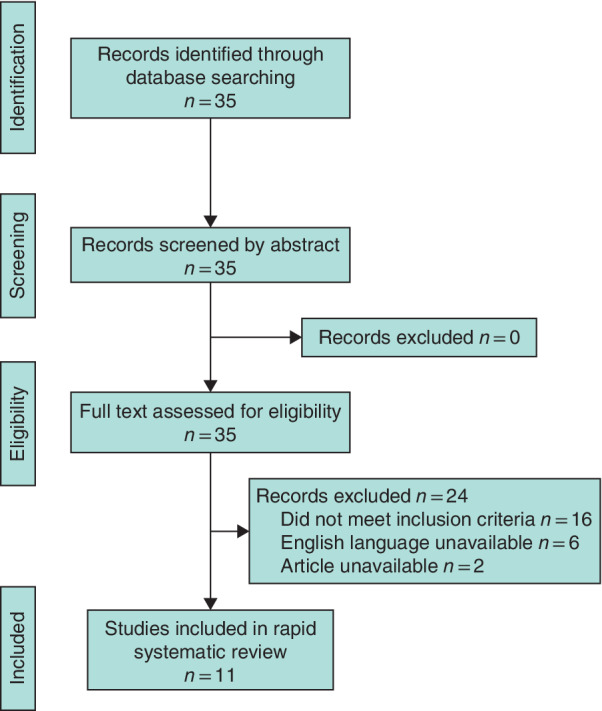
PRISMA diagram for the review

The MEDLINE search yielded a total of 35 articles. Full manuscripts were obtained for all of these, of which 11 articles^7^, ^8^, ^9^, ^10^, ^11^, ^12^, ^13^, ^14^, ^15^, ^16^, ^17^ met the eligibility criteria for inclusion. Nine^7^, ^9^, ^10^, ^11^, ^12^, ^13^, ^15^, ^16^, ^17^ of these 11 articles constituted expert opinion in the form of reviews or journal correspondence, and two^8^, ^14^ were observational studies, median OCEBM level 5 (range 4–5). All originated in the Far East (9 from China, 2 from Singapore), with the majority related to general surgery (8 articles). The inclusion pathway is illustrated in the PRISMA diagram (*Fig*. *1*).

*Table* *1* gives an overview of the evidence grading, and *Table* *2* outlines which of the five specified domains were covered in each paper.

**Table 1:** Characteristics of included studies

Reference	Country	Design	OCEBM level of evidence	Subspecialty	Cohort size
Tao et al.^7^	China	Expert opinion/experience	5	General surgery	–
Gou et al.^8^	China	Case report/series (retrospective)	4	HPB	4
Wong et al.^9^	Singapore	Expert opinion/experience	5	–	–
Ti et al.^10^	Singapore	Expert opinion/experience	5	–	–
Wu et al.^11^	China	Expert opinion/experience	5	HPB	–
Li et al.^12^	China	Expert opinion/experience	5	Upper GI	–
Luo and Zhong^13^	China	Expert opinion/experience	5	Colorectal	–
Chen et al.^14^	China	Case report/series (retrospective)	4	Obstetrics	17
Li et al.^15^	China	Expert opinion/experience	5	HPB	–
Hu et al.^16^	China	Expert opinion/experience	5	Colorectal	–
Chen and Peng^17^	China	Expert opinion/experience	5	Upper GI	–

OCEBM, Oxford Centre for Evidence‐Based Medicine; HPB, hepatopancreatobiliary; GI, gastrointestinal.

**Table 2:** Domains covered in each study

	Domain				
Reference	1	2	3	4	5
Tao et al.^7^	✓	✓	✓	✓	✓
Gou et al.^8^	✓	✓	✓	✓	
Wong et al.^9^	✓	✓	✓	✓	✓
Ti et al.^10^	✓	✓	✓	✓	✓
Wu et al.^11^	✓		✓	✓	✓
Li et al.^12^	✓	✓	✓	✓	✓
Luo and Zhong^13^	✓	✓	✓		✓
Chen et al.^14^	✓	✓	✓	✓	✓
Li et al.^15^	✓		✓	✓	✓
Hu et al.^16^	✓		✓	✓	✓
Chen and Peng^17^	✓	✓			✓

### Physical operating room factors

All 11 papers contained recommendations relating to the physical OR. Despite wide‐ranging specialty coverage, common themes for preparation of the OR were identified as shown in *Table* *3*.

**Table 3:** Domain 1: recommendations relating to physical operating room factors


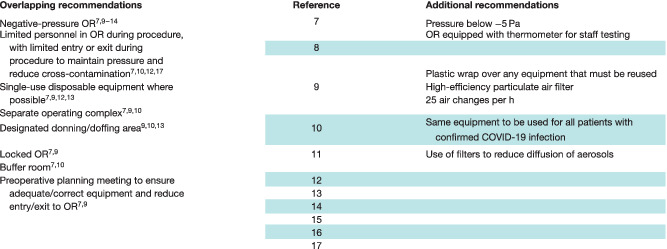

OR, operating room.

Both of the case series reported data from patients treated within a single centre. One study^14^ involved 17 pregnant women who tested positive for COVID‐19 and had a caesarean section in Renmin University Hospital, Wuhan, China. Recommendations for the physical OR specified the use of a negative‐pressure OR, alongside strict separation of clean and contaminated areas, and a buffer system to ensure that clean rooms remain as such, avoiding cross‐contamination. The other study^8^, from Tongji Medical College in Wuhan, also acknowledged and discussed the theme of separation of patients with COVID‐19 from those without the disease.

The remaining nine papers made suggestions derived from local operative experience during the pandemic. Use of a negative‐pressure OR, along with a number of other practices to minimize intraoperative viral load, was recommended in six^7^, ^9^, ^10^, ^11^, ^12^, ^13^ of these articles (*Table* *3*).

The use of single‐use/disposable equipment for COVID‐19‐positive patients was recommended in four communications^7^, ^9^, ^12^, ^13^. Other units recommended use of the same anaesthetic equipment for COVID‐19‐positive patients only^10^, or covering equipment with disposable plastic wrapping when required for use in multiple patients^9^. In two^7^, ^9^ of these papers, the need for adequate preoperative planning was highlighted, to ensure availability of equipment that may be required, and to minimize store cupboard visits and consequent risks of cross‐contamination and disturbance of the pressure within the OR. One article^11^, from Beijing National Cancer Centre, referred to the use of suction or filters to diffuse aerosols, but without offering specific instruction.

### Personnel factors

Of the 11 communications, eight^7^, ^8^, ^9^, ^10^, ^12^, ^13^, ^14^, ^17^ specified measures directly related to staff members involved in the operative care of patients (*Table* *4*). One^14^ reported that all staff involved in the operative care of COVID‐19‐positive pregnant patients undergoing caesarean delivery were swabbed after surgery to establish their own COVID‐19 status, and had thoracic CT every 2 weeks. The number of postoperative CT scans performed per member of staff and the level of preoperative screening undertaken was not reported. Appropriate personal protective equipment (PPE), including N95 or powered air‐purifying respirator (PAPR) masks, was used for all staff. Of the 38 staff involved in the care of these patients, none tested positive for the virus subsequently^14^.

**Table 4:** Domain 2: recommendations relating to personnel factors


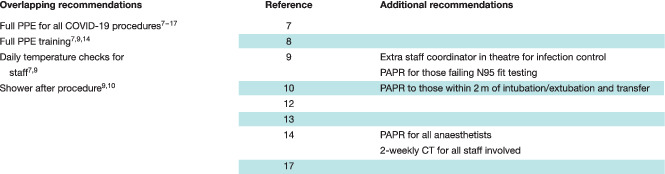

PPE, personal protective equipment; PAPR, powered air‐purifying respirator.

Indications for the use of PAPR masks varied (*Table* *4*), although full PPE, and formal training in its use, was recommended in all articles^7^, ^8^, ^9^, ^10^, ^11^, ^12^, ^13^, ^14^, ^15^, ^16^, ^17^ for patients with suspected or confirmed COVID‐19 infection. An extra staff coordinator in theatre, assigned to provide guidance in staff members' new roles and aid with unfamiliarity in new infection prevention procedures, was described at a tertiary centre in Singapore^9^.

Two articles^9^, ^10^ mandated staff showering after an operation, and two hospital units undertook temperature checks at least daily for all staff^7^, ^9^.

### Patient factors

Ten articles^7^, ^8^, ^9^, ^10^, ^11^, ^12^, ^13^, ^14^, ^15^, ^16^ highlighted patient‐related considerations (*Table* *5*). In patients for whom surgery was advocated, the importance of screening was a common theme; nine articles described practices ranging from questionnaires and swabs to thoracic CT^7^, ^8^, ^9^, ^11^, ^12^, ^13^, ^14^, ^15^, ^16^, and recommended self‐isolation before any elective admission^8^, ^11^, ^12^, ^13^, ^16^.

**Table 5:** Domain 3: recommendations relating to patient factors


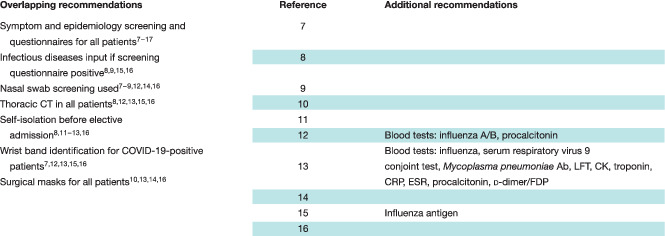

Ab, antibodies; LFT, liver function tests; CK, creatinine kinase; ESR, erythrocyte sedimentation rate; FDP, fibrin degradation products.

A host of routine blood tests were recommended by Li and colleagues^12^ and Luo and Zhong^13^ before hospital admission, which included procalcitonin, and both type A and type B influenza screening (*Table* *5*).

Four articles^10^, ^13^, ^14^, ^16^ recommended surgical masks for all patients, especially on transfer, with special routes and elevators used for infected patients. The use of wrist bands, issued to all patients testing positive for COVID‐19, was described in five articles^7^, ^12^, ^13^, ^15^, ^16^ as a method to promote ready identification.

Communication with patients and families was highlighted. Patients with cancer were described as needing special consideration, with four articles^7^, ^11^, ^12^, ^13^ recommending nutritional support, and two^11^, ^12^ additional psychological support.

### Procedural factors

Nine articles^7^, ^8^, ^9^, ^10^, ^11^, ^12^, ^14^, ^15^, ^16^ outlined OR procedural details. Of these, four^7^, ^9^, ^10^, ^14^ focused on technical adjustments to provide safer anaesthetic protocols. The remainder^8^, ^9^, ^11^, ^12^, ^15^, ^16^ reported modifications made to surgical planning or interventions.

Seven focused on individual subspecialty practice, including preoperative considerations. Although each of these concentrated on different surgical circumstances, overlapping themes were apparent, as described in *Table* *6*. A common theme was to avoid surgery where possible. In the emergency setting, temporizing measures such as stenting, endoscopic drainage or embolization were considered alternatives. Three articles^7^, ^9^, ^16^ recommended that emergency patients testing negative for COVID‐19 could be treated as normal and undergo surgery if necessary. No study had been published focusing solely on the management of general surgical emergencies in the OR during the COVID‐19 crisis.

**Table 6:** Domain 4: recommendations relating to anaesthetic and surgical procedures (procedural factors)


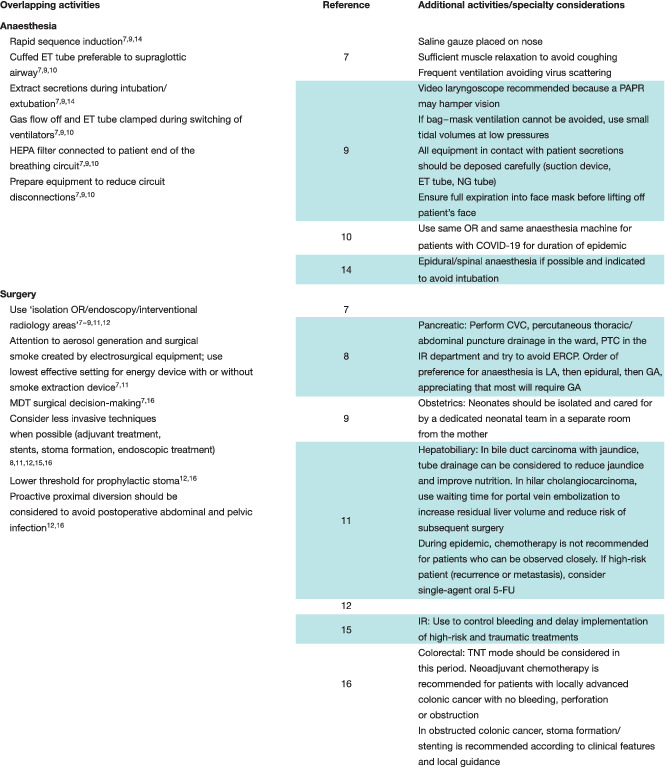

ET, endotracheal; PAPR, powered air‐purifying respirator; HEPA, high‐efficiency particulate air; NG, nasogastric; OR, operating room; CVC, central venous catheter; PTC, percutaneous transhepatic cholangiography; IR, interventional radiology; ERCP, endoscopic retrograde cholangiopancreatography; LA, local anaesthesia; GA, general anaesthesia; MDT, multidisciplinary team; 5‐FU, 5‐fluorouracil; TNT, total neoadjuvant therapy.

### Other considerations

The main additional area considered was postoperative management. Most described the importance of altered postoperative practice, eight articles^7^, ^8^, ^9^, ^12^, ^13^, ^14^, ^15^, ^16^ recommending limiting visitors, with any visitors regularly checked for fever (*Table* *7*). Six articles^7^, ^9^, ^11^, ^13^, ^15^, ^16^ recommended limiting in‐person contact with physicians, replacing this with video calls where possible.

**Table 7:** Domain 5: recommendations relating to other considerations


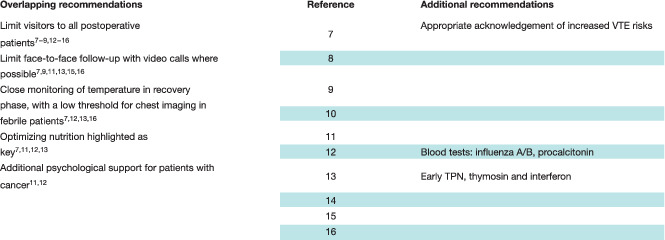

VTE, venous thromboembolism; TPN, total parenteral nutrition.

Recognizing the risks of significant complications, four articles^7^, ^12^, ^13^, ^16^ describing practice in general surgery recommended that any postoperative pyrexia should be considered an indication for chest CT to look for possible COVID‐19 infection.

Nutrition was again highlighted in four articles^7^, ^11^, ^12^, ^13^, one of which advised early parenteral nutrition, supplemented with thymosin and interferon^13^. A single communication^7^ specifically raised the risk of venous thromboembolism in the postoperative period, given the increased risks associated with reduced mobility in patients with COVID‐19.

## Discussion

This systematic review identified and evaluated evidence of OR best practice in the face of the COVID‐19 pandemic. The research question was deliberately kept broad, to optimize the volume of clinical practice and consequent literature reports available. At the time of the review (19 March 2020), COVID‐19 had been reported in 156 countries^18^.

Although the epidemiology of COVID‐19 has been well reported^19^, ^20^, this systematic review found little robust evidence regarding safe and best surgical practice. Nine of the 11 papers provided OCEBM level 5 evidence, generating grade D recommendations consistent with very low quality and inconclusive evidence^6^. Consensus regarding perioperative practice was apparent, from institutions exposed to high COVID‐19 caseloads. Within the operating suite, common recommendations focused on room design, in particular geographic segregation of COVID‐19‐positive areas and the use of negative‐pressure ventilation. Such practices were reported previously^21^, ^22^ during the severe acute respiratory syndrome (SARS) and Middle East respiratory syndrome (MERS) epidemics, in an attempt to minimize the risk of cross‐contamination from areas of negative to normal air pressure. Evidence regarding actions to minimize viral load was sparse, but such general measures were pragmatic and achievable in most, if not all, hospitals.

The importance of appropriate PPE, both before and during surgery, to protect staff and minimize viral transmission was highlighted in all articles, supported by evidence in reports from previous outbreaks of SARS and MERS^23^, ^24^, ^25^. Other studies have emphasized the importance of adequate training in the correct use of PPE, where poor protocol compliance has been shown to have a significant association with viral transmission^26^, ^27^. In the present review, guidance regarding the use of PAPR and N95 masks was inconsistent, with only two^10,14^ of the 11 articles mandating the use of PAPR during high‐risk procedures such as intubation. The filtering ability of PAPR masks is reported to be marginally better than that of N95 masks (99 *versus* 95 per cent respectively filtration of particles smaller than 5 μm in diameter), but the clinical significance of this is unknown^28^.

The level of screening necessary for surgical patients differed between hospital units. All recommended health questionnaires or nasopharyngeal swabs as a minimum requirement, with additional thoracic CT recommended by almost half. Evidence‐based consensus regarding patient screening is therefore a priority, based on the balance of risk and sensitivity of individual screening test modalities.

The issue of a deliberate pause in elective surgical practice was a common theme, with the justification cited being to minimize further viral transmission, strategically to ration a stretched health resource threatened with an overwhelming burden, and as a response to the risk of unknown potential adverse clinical outcomes of patients developing COVID‐19‐related morbidity and complications after surgery. To mitigate potential surgical and respiratory morbidity, consideration of less invasive procedures such as interventional radiology or endoscopy was recommended, and in those requiring surgical resection the formation of proximal diversion or end stomas to avoid the risk of operative sepsis associated with anastomotic leakage^8^, ^11^, ^12^, ^15^, ^16^. Such recommendations seem reasonable and have subsequently been published in guidance documents produced by international professional associations^29^, ^30^, ^31^.

Minimizing intraoperative aerosol generation and the potential for dissemination of viral particles was addressed in a number of the papers included in this review. The use of rapid sequence induction with cuffed endotracheal tubes and limiting energized dissection, as reported, are recognized techniques in the literature; however, high‐quality evidence‐based practices are lacking^32^, ^33^, ^34^, ^35^, ^36^, ^37^.

There are several limitations to this review. The number of publications available for review was modest. Rapid evaluation of evidence, which has been essential in informing early recommendations in an emerging pandemic, can result in false assumptions and conclusions. The results are subject to the risk of significant bias. The study relied on the inclusion of expert opinion and low‐quality observational studies, owing to the absence of high‐quality clinical trials. In exceptional circumstances, such as the current coronavirus pandemic, a conventional strong evidence‐based approach to healthcare policy development is neither feasible nor safe.

Within the constraints of time and resource, clinical practice must be driven by a pragmatic approach to developing evidence‐based practice, and arguably a novel scientific approach to collating global evidence when rigorous research evidence is not available. The widespread professional use of social networking platforms represents a powerful way of sharing such information internationally.

This review has highlighted the need for novel methodological approaches. There is a need to assimilate ‘real‐time’ preliminary experiences and evidence, in advance of publication, while recognizing the heightened potential for bias. An ability to transcend geographical and language barriers is essential to facilitate international knowledge‐sharing. The encouragement of wide participation across a range of disciplines, not limited to healthcare professionals, would help to capture the full spectrum of viewpoints and potential solutions. Finally, the ability to recruit a pool of engaged stakeholders, ready to assist, would permit iterative development and re‐evaluation as a situation unfolds.

## Collaborators

Members of the WSRI Collaborative: T. Abdelrahman, J. Ansell, C. Brown, R. Egan, T. Evans, E. Ryan Harper, R. L. Harries, L. Hopkins, O. James, S. Lewis, W. G. Lewis, O. Luton, K. Mellor, A. G. Powell, D. Robinson, R. Thomas, A. Williams, A. J. Beamish.

## Supporting information


**Appendix S1**: Supporting information
