# Supplementary material for: Recommended operating room practice during the COVID‐19 pandemic: systematic review
Source: BJS Open. 2020 Jun 4;4(5):748–56. doi: 10.1002/bjs5.50304 (PMC7272923; doi:10.1002/bjs5.50304)
Supplement: Supplementary file 1 — Appendix S1: Supporting information [file BJS5-4-748-s001.docx]

**BJS5_50304**

**Recommended operating room practice during the COVID-19 pandemic: systematic review**

**Welsh Surgical Research Initiative (WSRI) Collaborative**

**Appendix S1** Review search algorithm

| coronavirus OR novel coronavirus OR covid OR covid19 OR covid-19 OR SARS-CoV-2 OR SARS CoV 2  AND  operating room OR theatre OR surgery OR operation  AND  preparation OR consideration OR measures OR precautions OR procedures OR guidance) OR guideline OR advice OR advise OR recommend OR practice |
| --- |
